# ^23^Na and ^27^Al NMR Study of Structure and Dynamics in Mordenite

**DOI:** 10.1007/s00723-016-0847-8

**Published:** 2016-11-09

**Authors:** N. A. Sergeev, M. Paczwa, M. Olszewski, A. M. Panich

**Affiliations:** 1 Institute of Physics, University of Szczecin, 70-451 Szczecin, Poland; 2 Department of Physics, Ben-Gurion University of the Negev, P. O. Box 653, 8410501 Be’er Sheva, Israel

## Abstract

Temperature dependencies of ^27^Al and ^23^Na nuclear magnetic resonance spectra and spin–lattice relaxations in mordenite have been studied in static and magic angle spinning regimes. Our data show that the spin–lattice relaxations of the ^23^Na and ^27^Al nuclei are mainly governed by interaction of nuclear quadrupole moments with electric field gradients of the crystal, modulated by translational motion of water molecules in the mordenite channels. At temperatures below 200 K, the dipolar interaction of nuclear spins with paramagnetic impurities becomes an important relaxation mechanism of the ^23^Na and ^27^Al nuclei.

## Introduction

**Fig. 1:**
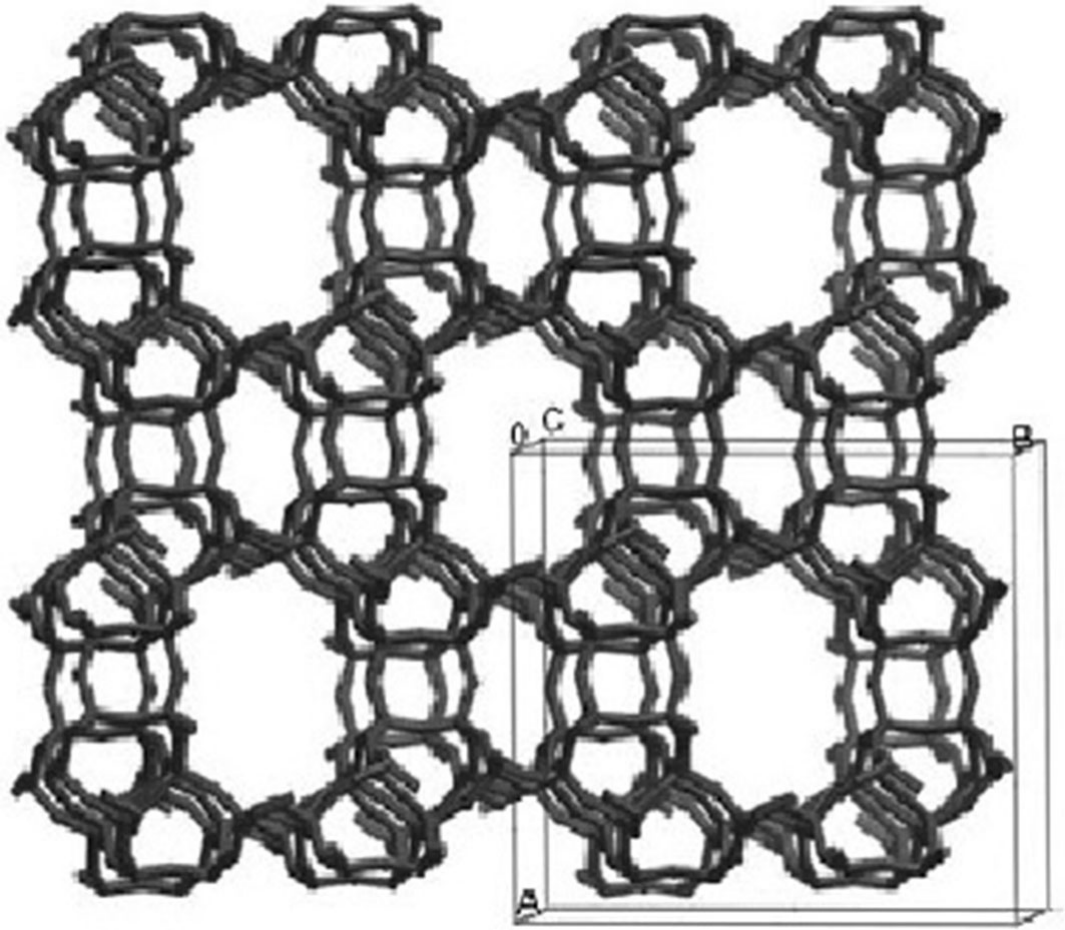
Framework structure of mordenite viewed along the 12-ring channels. The very small and elongated eight-ring channels can also be observed. The eight-ring channels running perpendicular to the large pores are not discernible in this picture

The mineral mordenite, Na_8_Al_8_Si_40_O_96_·24H_2_O, is a channel-type zeolite [1]. Its framework consists of AlO_4_ and SiO_4_ tetrahedra linked together via common oxygen atoms (Fig. 1). The mordenite structure exhibits two types of elliptical nanosized channels, both running along the *c*-axis [2, 3]. The wide channels have aperture of 0.65 × 0.70 nm and are formed by the assemblage of 12-membered rings, each having 12 oxygen atoms. The narrow channels have aperture of 0.26 × 0.57 nm and are built of eight-membered rings, each having eight oxygen atoms. The wide and narrow channels are linked by eight-membered ring channels (aperture 0.34 × 0.48 nm) running along the *b*-axis [2, 3]. Water molecules and Na^+^ ions are located in the above-mentioned channels [1–3] and possess certain mobility at ambient conditions. While water molecules can be desorbed from the channels, the sodium ions can be substituted by some other ions by means of ionic exchange [1].

The dynamics of water molecules in mordenite have been studied by nuclear magnetic resonance (NMR) [4, 5]. Analysis of the temperature dependencies of the proton spin-relaxation times in the laboratory and rotating frames (*T*_1_ and *T*_1ρ_) revealed diffusion of water molecules along the channels above *T* = 200 K. Temperature dependencies of the ^1^ *H* dipolar spin-relaxation time *T*_1D_ showed that the dipolar relaxation in mordenite is responsive to slow 180° reorientations (flips) of water molecules around their second-order symmetry axes.

In the present paper, we report on the NMR study of quadrupolar ^27^Al and ^23^Na nuclei in mordenite. We studied temperature dependencies of the ^27^Al and ^23^Na NMR spectra in static and magic angle spinning (MAS) regimes, as well as the temperature dependencies of the spin–lattice relaxation times of the ^27^Al and ^23^Na nuclei.

## Experiment

We studied a powder sample of natural mordenite from Nidym, Siberia, with chemical composition Na_0.9_Ca_0.05_AlSi_5_O_12_.3·1H_2_O [4]. The ^27^Al and ^23^Na NMR spectra were measured using a Bruker Avance-400 NMR spectrometer at resonance frequencies of 104.26652 and 105.842 MHz, respectively, in the applied magnetic field of *B*_0_ = 9.4 T. The MAS rate used in the experiments was 14 kHz. Free induction decay (FID) signals were recorded after applying a single radio-frequency (RF) pulse and 100 signal acquisitions. The NMR spectra of ^23^Na and ^27^Al were obtained by Fourier transformation of the FID signals. Both ^27^Al (natural abundance 100%, spin *I* = 5/2) and ^23^Na (natural abundance 100%, spin *I* = 3/2) are quadrupolar nuclei, and their NMR spectra consist of the central transition (+1/2 ⟷ −1/2) and a number of satellites. For a selective excitation of the central transition, the optimal pulse duration is equal to that of a non-selective π/2 pulse divided by (*I* + 1/2) [6]. In our experiments, the RF pulse of 1.0 μs was used. The spin–lattice relaxation times *T*_1_ of ^23^Na and ^27^Al nuclei were measured by saturation recovery method. The relaxation processes for ^23^Na and ^27^Al nuclei in mordenite are found to be well described by single exponentials. The Dmfit program [7] was used to simulate the ^27^Al spectra to extract the isotropic chemical shifts (*δ*_iso_), quadrupolar coupling constants (QCC), and the asymmetry parameters (*η*_*Q*_).

## Results and Discussion

The ^27^Al NMR spectrum of the powder mordenite is represented by a single line with isotropic chemical shift *δ*_iso_ of ~61 ppm. This value is characteristic of ^27^Al nuclei of AlO_4_ groups in different zeolites, in which *δ*_iso_ varies in the range of 60 ÷ 63 ppm [1, 8–11]. The lineshape of the quadrupolar ^27^Al nuclei in mordenite is determined by two contributions, namely by (1) the interaction of nuclear quadrupole moments with electric field gradients (EFG) on the ^27^Al sites and (2) nuclear dipolar interactions. To determine the contributions of different interactions to the NMR shape of ^27^Al in mordenite, we measured the ^27^Al NMR spectra with and without ^1^H decoupling and with and without sample spinning at different temperatures.

**Fig. 2:**
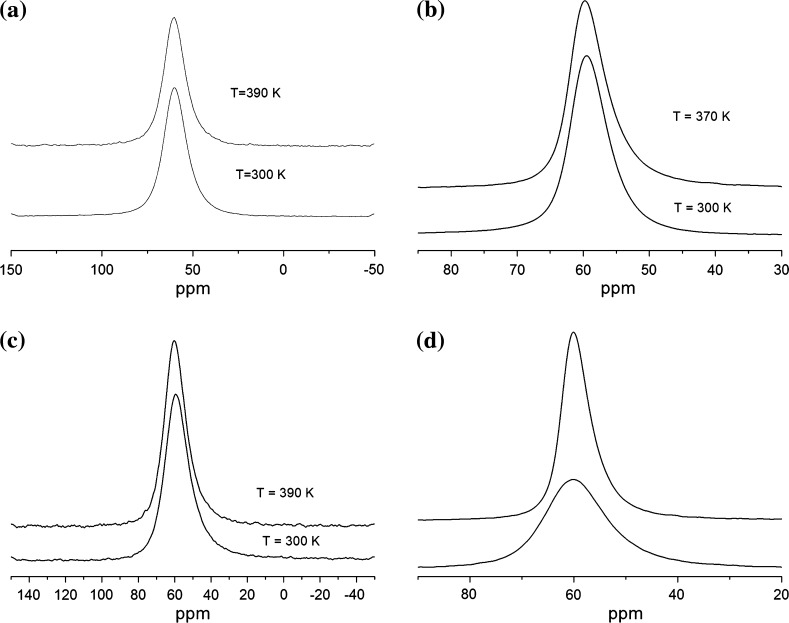
**a** ^27^Al NMR spectra with ^1^H-decoupling at *T* = 300 and *T* = 390 K; **b** ^27^Al MAS NMR spectra at *Ω*_rot_ = 14 kHz at *T* = 300 K and *T* = 370 K; **c** ^27^Al NMR spectra without ^1^H-decoupling at *T* = 300 K and *T* = 390 K; **d** ^27^Al MAS NMR spectra at *T* = 370 K; *Ω*_rot_ = 14 kHz (*top*) and *Ω*_rot_ = 0 kHz (*bottom*)

The ^27^Al NMR spectra of mordenite obtained at *T* = 300 K and *T* = 390 K are shown in Fig. 2. Figure 2a shows the spectra obtained using the ^1^H decoupling, which allows to eliminate the effect of the magnetic dipolar coupling between the resonance nuclei (in our case ^27^Al) and ^1^H nuclei. Herewith, the ^27^Al NMR spectra reveal the same shape and width (Δ*ν* = 1.5 kHz) at *T* = 300 and 390 K (Fig. 2a). Since the dipolar interactions between ^1^H and ^27^Al nuclei are averaged out by the ^1^H decoupling and thus only quadrupole interactions are the case, the aforementioned finding means that the temperature variation of electric field gradient (EFG) at the ^27^Al sites in the region of 300–390 K is slight and actually is not observed in the experiment.

The EFG at the ^27^Al site in the mordenite structure is caused by electric charges of the ions of crystal lattice and by the electric dipolar moments of water molecules. If the latter contribution is considerable, its averaging must be observable in the temperature dependence of ^27^Al NMR spectra. However, Fig. 2b, c does not show noticeable variations in the temperature range from 300 to 390 K. This means that the contribution of electric dipolar moments of water molecules to the static and MAS ^27^Al NMR line shapes at 300–390 K is very small. The same conclusion follows from the comparison of the ^27^Al NMR spectra obtained at *T* = 300 and 390 K measured with and without ^1^H decoupling, which are shown in Fig. 2a, c. These spectra show the same lineshape and width, indicating that the dipolar interactions between of ^27^Al and ^1^H nuclei are very small in the range from 300 to 390 K. This finding is in agreement with the flipping and diffusion of water molecules along the mordenite channels obtained by ^1^H NMR [5], which results in an effective averaging of the above-mentioned contribution at *T* > 300 K.

The rotation of sample at the magic angle leads to complete averaging of the dipolar interaction among ^27^Al and other nuclei (^1^H, ^23^Na and, ^29^Si) in mordenite. In this case, the shape of NMR spectra is determined only by the incompletely averaged by MAS second-order quadrupole effects contributing to the central transition [6].

The second moment of the powder MAS NMR spectrum is defined by equations [6]:

1 $$M_{2}^{\text{MAS}} = 0.25 \times \nu_{\text{iso}}^{2}$$

2 $$\nu_{\text{iso}} = - \frac{{\nu_{Q}^{2} }}{{30\nu_{L} }}\left[ {I\left( {I + 1} \right) - \frac{3}{4}} \right]\left( {1 + \frac{{\eta^{2} }}{3}} \right).$$

Here, the quadrupole frequency is

3 $$\nu_{Q} = \frac{{3e^{2} qQ}}{2I(2I - 1)h} \equiv \frac{3}{2I(2I - 1)}C_{\text{qcc}} ,$$

where *ν*_*L*_ is the Larmor frequency, *q* is the electric field gradient, *η* is the asymmetry parameter of EFG, *Q* is the nuclear quadrupole moment, and $C_{\text{qcc}} = \frac{2I(2I - 1)}{3}v_{Q} \equiv \frac{{e^{2} qQ}}{h}$ is the quadrupole coupling constant (QCC) [6].

^27^Al NMR spectra at *T* = 370 K shown in Fig. 2d reveal noticeable difference in static and MAS regimes, i.e., line width Δ*ν* = 1.53 and 0.74 kHz at *Ω*_rot_ = 0 and 14 kHz, respectively. These spectra yield $M_{2}^{\text{static}} \approx 2.4\,{\text{kHz}}^{2}$ and $M_{2}^{\text{MAS}} \approx 0.2\,{\text{kHz}}^{2}$, thus $\sqrt {\frac{{M_{2}^{\text{static}} }}{{M_{2}^{\text{MAS}} }}} \approx 3.5$, which is close to the ratio of 3.6 calculated in [6]. Using Eqs. (1–3), we obtain $\nu_{\text{iso}} \approx 0.9\,{\text{kHz}}$, $v_{Q} \sqrt {1 + \frac{{\eta^{2} }}{3}} \approx 0.6\,{\text{MHz}}$, and $C_{\text{qcc}} \sqrt {1 + \frac{{\eta^{2} }}{3}} \approx 4\,{\text{MHz}} .$

**Fig. 3:**
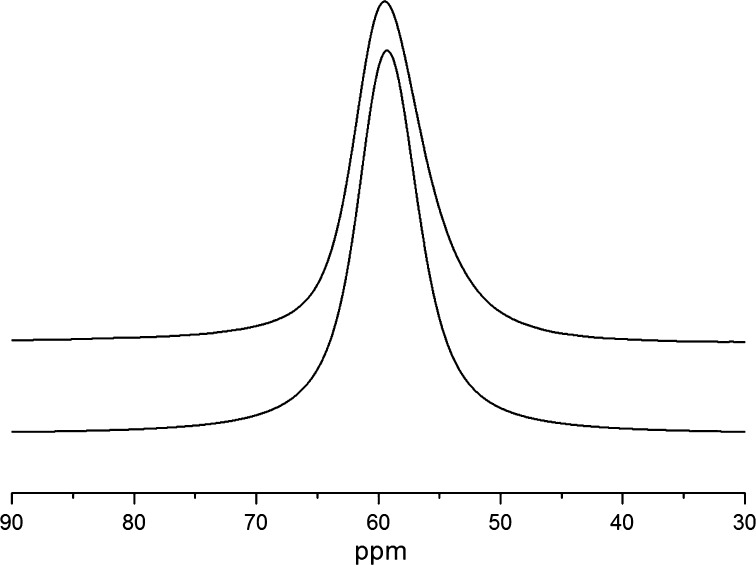
Experimental ^27^Al MAS NMR spectrum of mordenite measured at *T* = 300 K and *Ω*_rot_ = 14 kHz (*bottom*) and the simulated one for *C*_qcc_ = 2 (0.2) MHz, *η* = 0.5 (0.1), *δ*_*CSA*_ = 61.21 (0.07) ppm, and Δ*ν*_Lorentz_ = 460.5 (0.5) Hz

The absence of the fine structure in ^27^Al NMR spectra (Fig. 3) does not allow us to precisely determine parameters *C*_qcc_ and η. Using the DMFit program [7], we simulated the shape of the powder ^27^Al MAS NMR spectrum in mordenite and found the optimal values of *C*_qcc_ = 2 (0.2) MHz and *η* = 0.5 (0.1), as shown in Fig. 3.

The literature data [1, 6, 8–11] show the QCC (^27^Al) *C*_qcc_ in different zeolites are in the range of 2 ÷ 5 MHz. One can find that our experimental values are within this range. We note that Chae et al. [12] calculated the quadrupole parameters of Al in mordenite with various Si/Al rations using the point change model [13] with the 4 and 20 nearest neighbor oxygen atoms. The coordinates of the atoms surrounding Al sites were taken from the neutron powder diffraction data [14]. However, the obtained value *C*_qcc_ ≤ 1 MHz is different from the experimental one obtained in this paper.

**Fig. 4:**
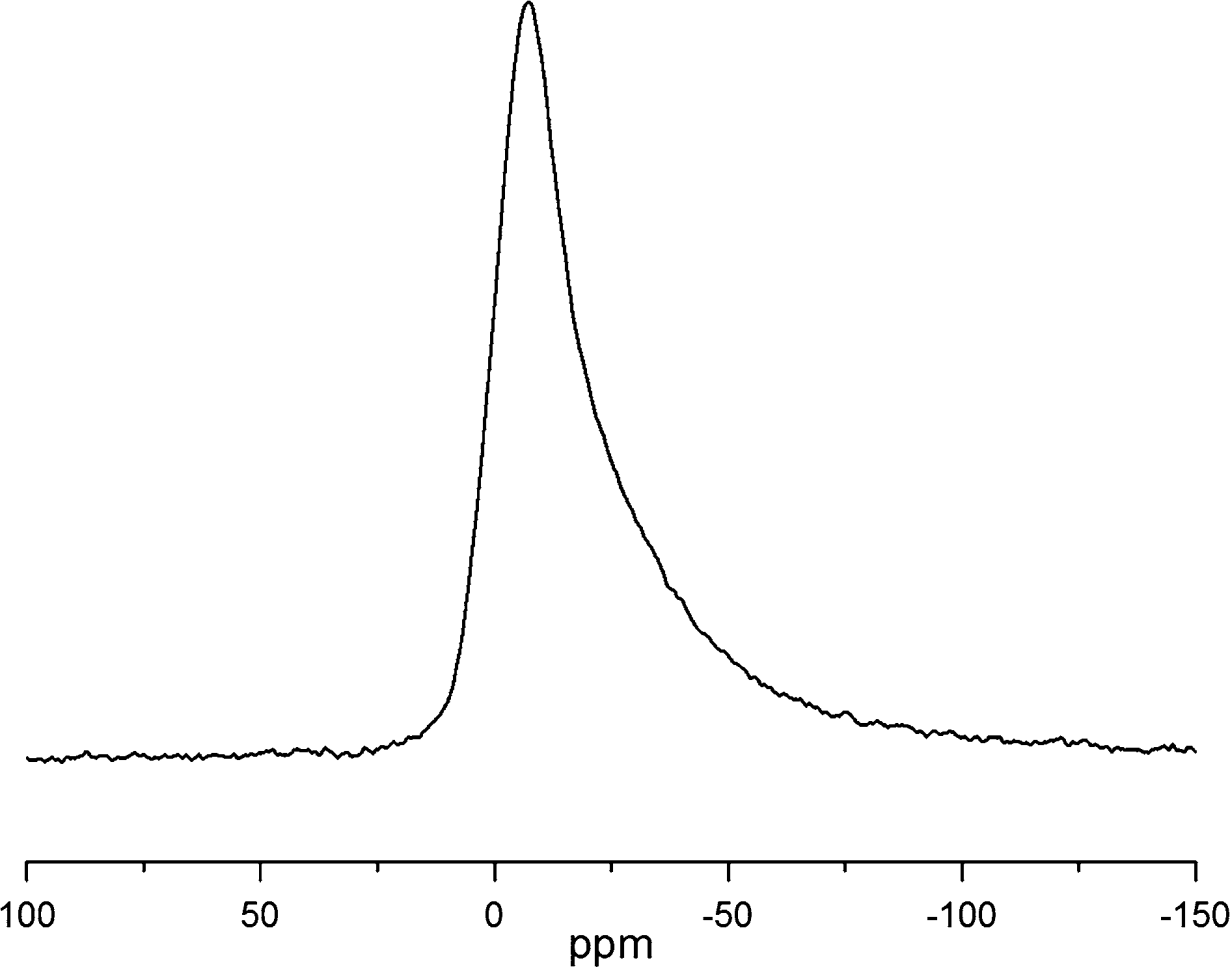
Experimental ^23^Na MAS NMR spectrum of mordenite at *T* = 200 K and MAS 14 kHz

The sodium cations in the mordenite structure are mainly located in eight-membered oxygen rings, occupying positions away from the channel center. Some of the cations are also placed in the 12-ring channels [1–3]. The magic angle spinning of the sample results in complete averaging of dipolar interaction of ^23^Na spins with the spins of the other nuclei in mordenite, i.e., with ^1^H, ^23^Na, ^29^Si, and ^27^Al. In the case that all sodium nuclei are equivalent, the shape of the central NMR transition would be determined only by the incompletely averaged by MAS second-order quadrupole interaction term [8], which yields a characteristic asymmetric line. Such asymmetric line is observed in the experimental ^23^Na MAS NMR spectrum of mordenite at *T* = 200 K (Fig. 4). In the case in question, however, such lineshape may also be caused by structurally inequivalent sodium ions located in the wide and narrow channels of mordenite. We note that similar asymmetric line was observed by Hunger et al. [15] in one-dimensional ^23^Na MAS NMR spectrum of dehydrated mordenitie, while their two-dimensional triple-quantum (2D-3Q) ^23^Na MAS NMR spectrum revealed two peaks, attributed to sodium cations located in the side pockets of eight-membered oxygen rings and those on positions in the 12-ring channels. As known, mordenite dehydration leads to some displacements of the cations making their structural positions in dehydrated and non-dehydrated mordenites somewhat different. Anyhow, taking into account an inequivalence of the sodium ion positions, it is worth suggesting that the ^23^Na line asymmetry observed is caused by two contributions, namely, by the quadrupole interaction and a superposition of NMR signals coming from the structurally inequivalent sodium atoms with two different populations.

**Fig. 5:**
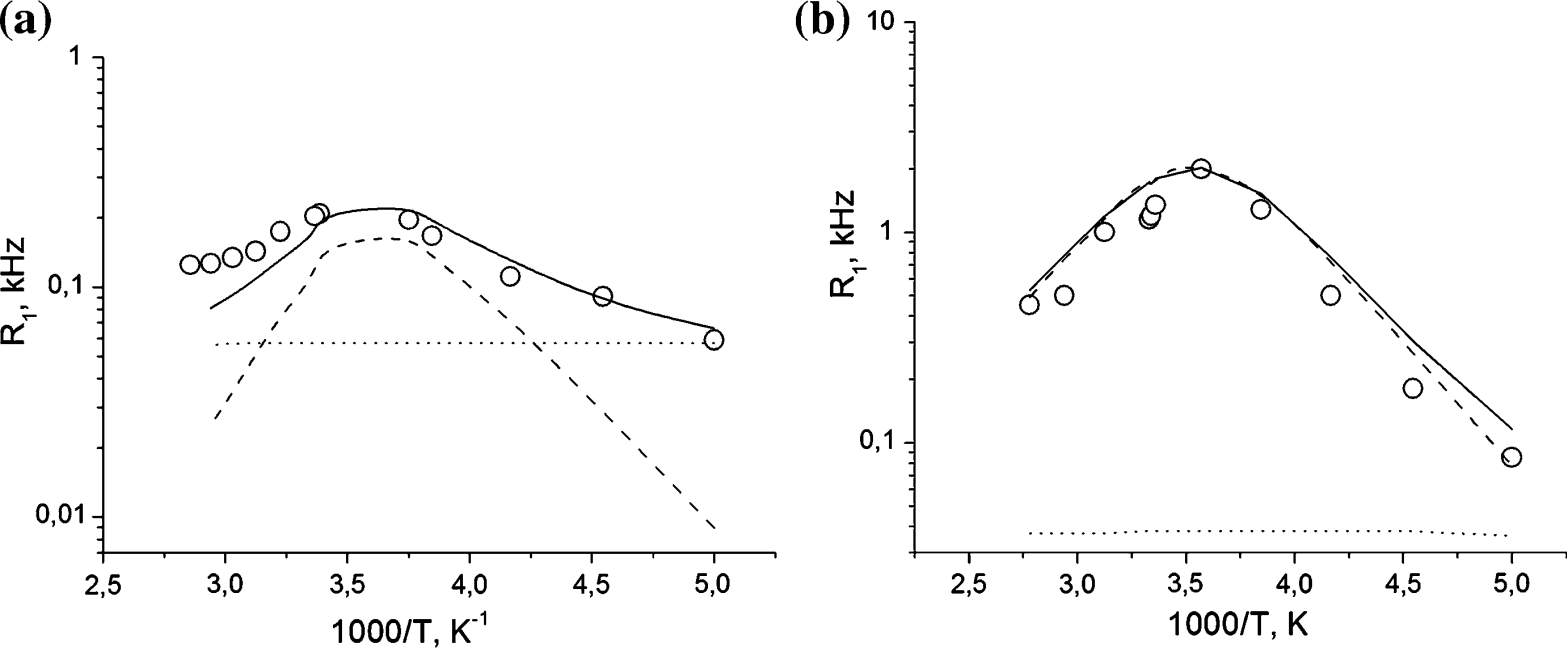
Temperature dependencies of the spin–lattice relaxation rates of ^27^Al (**a**) and ^23^Na (**b**) nuclei in mordenite. Experimental data are shown by *open circles*, and simulated contributions of quadrupole interactions and paramagnetic impurities are shown by *dashed* and *dotted lines*, correspondingly

The temperature dependencies of the ^27^Al and ^23^Na spin–lattice relaxation rates *R*_1_ in mordenite are shown in Fig. 5.

There are several contributions to the longitudinal relaxation of the ^23^Na and ^27^Al nuclei [16], namely, (1) interaction of the nuclear quadrupole moment with electric field gradient of the crystal modulated by lattice vibrations (spin–phonon interaction) and motion of cations and water molecules, (2) magnetic dipolar interaction of nuclear spins with paramagnetic impurities, and (3) magnetic dipolar interaction with magnetic moments of the framework ions (^27^Al, ^29^Si, ^23^Na) and of ^1^H nuclei of water molecules [16]. Herewith, cation and water motions are of a hopping-like nature, i.e., these atoms and molecules spent most of time in potential wells corresponding to the equilibrium positions, while the time of molecular jumps between these potential wells is much shorter.

The phonon-based relaxation mechanism is known to be too slow and leads to the relaxation times being by 4–5 orders of magnitude longer than those experimentally obtained for quadrupolar nuclei in zeolites [16]. The spin–lattice relaxation via paramagnetic impurities usually dominates at low temperatures [16] that are beyond the temperature range of our study. Our estimation of the contribution of ^1^H–^27^Al and ^1^H–^23^Na magnetic dipolar interactions to the ^27^Al and ^23^Na spin–lattice relaxations yield *T*_1min_ that is much longer than the experimental values of *T*_1min_ varying from 0.5 to 5 s (Fig. 5). Therefore, in the case in question, only quadrupolar mechanism needs to be considered.

For nuclei with *I* > 1/2, such as ^27^Al (*I* = 5/2) and ^23^Na (*I* = 3/2), the energy-level spacings in magnetic field are rendered unequal by quadrupole interaction, and the nuclear spin–lattice relaxation process is described by 2*I* relaxation times [15–20]. However, usually, the difference between these exponentials in such a multiexponential process can hardly be distinguished and, particularly for selective excitation of the central transition [6], the relaxation is well described by an exponential function [16, 21–25]. This is the case of our experimental ^27^Al and ^23^Na relaxation data in mordenite, which are well described by a single exponential.

Translational and orientational diffusion of water molecules modulates only a part of the EFG tensor governed by electric dipolar moments of water molecules, while the other part of the EFG tensor determined by the electric charges of the ions of lattice remains unchanged. The quadrupolar relaxation related to the former mechanism is described as [15–20]

4 $$R_{1Q} \equiv T_{1Q}^{ - 1} \cong \alpha \times \overline{{\left(\delta{{C}_{\text{Q}}} \right)}}^{2} \left[ {\frac{{\tau_{c} }}{{1 + \omega_{0}^{2} \tau_{c}^{2} }} + \frac{{4\tau_{c} }}{{1 + 4\omega_{0}^{2} \tau_{c}^{2} }}} \right],$$

where *ω*_0_ = 2*πν*_*L*_ is the Larmor frequency of the quadrupole nucleus, $\delta C_{Q} = \frac{{e^{2} \left( {\delta q} \right)Q}}{\hbar }$ describes the fluctuations of the quadrupole coupling constant [20] at the resonant nuclei sites, and $\tau_{c}$ is the correlation time of activated translational and reorientational jumps of electric dipoles of water molecules. Parameter α depends on the asymmetry parameter *η* and was estimated by us as *α* ≈ 0.13 ÷ 0.17. The symbol $\overline{\left( \cdots \right)}$ represents an ensemble averaging.

Equation (4) yields $R_{1Q} \sim \tau_{c}$ for $\omega_{0} \tau_{c} < < 1$ and $R_{1Q} \sim {1 \mathord{\left/ {\vphantom {1 {\tau_{c} }}} \right. \kern-0pt} {\tau_{c} }}$ for $\omega_{0} \tau_{c} > > 1$ with a maximum of $R_{1Q}$ (corresponding to the minimum of $T_{1Q}$) in the intermediate region. Since the correlation time *τ*_*c*_ caused by the molecular motion usually follows the Arrhenius-type temperature dependence, $\tau_{c} = \tau_{0} \exp \left( {{{E_{a} } \mathord{\left/ {\vphantom {{E_{a} } {\text{RT}}}} \right. \kern-0pt} {\text{RT}}}} \right)$, the asymptotic behavior of $\log R_{1Q} ({1 \mathord{\left/ {\vphantom {1 T}} \right. \kern-0pt} T})$ is represented by a straight line. Just such a behavior is obtained in our experiment in the temperature range from 200 to 390 K (Fig. 5). The deviation from the linear slowdown below *T* = 200 K is known to be caused by the interaction of nuclear spins with paramagnetic defects and impurities [18, 25, 26]. This contribution, mediated by the dipole–dipole interaction of nuclear spins with unpaired electron spins of paramagnetic defects, is given by expression [25–30]:

5 $$R_{1ne} \equiv T_{1ne}^{ - 1} = \gamma^{2} \left\langle {H_{L}^{2} } \right\rangle \frac{{\tau_{ce} }}{{1 + (\omega_{0} \times \tau_{ce} )}},$$

where $\left\langle {H_{L}^{2} } \right\rangle$ is determined as

6 $$\left\langle {H_{L}^{2} } \right\rangle = \frac{2}{5}\mu_{p}^{2} \frac{{N_{p} }}{N}\sum\limits_{i} {r_{ij}^{ - 6} } .$$

where $\mu_{p}^{2} = J(J + 1)\gamma_{J}^{2} \hbar^{2}$ is the squared magnetic moment of the paramagnetic defect, *N*_*p*_ is the density of the paramagnetic defects, *N* is the density of the resonant atoms, $\tau_{ce}$ is the correlation time that describes the reorientation of the electron spin caused by electron spin–lattice relaxation, and *r*_*ij*_ is the distance from the *j*th paramagnetic center to the *i*th resonant nucleus. Therefore, the experimental temperature dependences of the spin–lattice relaxation rates in the mordenite are described by expression:

7 $$R_{1} = T_{1}^{ - 1} = \alpha \times \overline{{\left( {{\delta{C}}_{\text{Q}} } \right)}}^{2} \times \left[ {\frac{{\tau_{c} }}{{1 + \left( {\omega_{0} \tau_{c} } \right)^{2} }} + \frac{{4\tau_{c} }}{{1 + \left( {2\omega_{0} \tau_{c} } \right)^{2} }}} \right] + \gamma^{2} \left\langle {H_{L}^{2} } \right\rangle \frac{{\tau_{ce} }}{{1 + \left( {\omega_{0} \tau_{ce} } \right)^{2} }}.$$

The experimental temperature dependencies of the spin–lattice relaxation rates and the results of their simulation by function of Eq. (7) are presented in Fig. 5. The obtained adjusting parameters are given in Tables 1 and 2. Figure 5 shows a satisfactory agreement between the experimental data and calculations.

**Table 1:** Calculated parameters that describe contribution $R_{1Q} \equiv T_{1Q}^{ - 1}$ to the spin–lattice relaxation of ^27^Al and ^23^Na nuclei in mordenite

Nucleus	$\alpha {\overline{{\left(\delta {{C}_{\text{Q}} } \right)}}^{2}} \times 10^{4}$, (rad kHz)^2^	*τ*_0_, s	$\bar{E}_{a}$, kJ/mol
^27^Al	7.9 (0.7)	10^−13^	21.6 (0.5)
^23^Na	266 (70)	10^−13^	22.7 (0.5)

**Table 2:** Calculated parameters that describe contribution $R_{1ne} = T_{ne}^{ - 1}$ to the spin–lattice relaxation of ^27^Al and ^23^Na nuclei in mordenite

Nucleus	$\gamma^{2} \left\langle {H_{L}^{2} } \right\rangle \times 10^{4}$, (rad kHz)^2^	$\tau_{0e}$, s	$E_{e1}$, kJ/mol
^27^Al	7.5 (0.8)	10^−9^	0.8 (0.1)
^23^Na	5.0 (0.5)	10^−9^	0.8 (0.1)

The obtained activation energy of ~21 kJ/mol suggests that the modulation of quadrupole interactions by means of translational and reorientational jumps of electric dipoles of water molecules [4, 5] is responsible for the relaxation process of the ^27^Al and ^23^Na nuclei. From the data represented in Table 1, the averaged fluctuations of the quadrupole coupling constants are found to be $\sqrt {\overline{{\left(\delta {{C}_{\text{Q}} } \right)}}^{2} \approx 0.03\,{\text{MHz}}}$ for ^27^Al nuclei and $\sqrt {\overline{{\left( \delta{{C}_{\text{Q}} } \right)}}^{2} \approx 0.16\,{\text{MHz}}}$ for ^23^Na nuclei.

Unfortunately, the absence of reliable coordinates of hydrogen atoms and discrepancies in the published coordinates of the other atoms in the mordenite structure does not allow estimation of the contributions of various atoms to EFG. Our rough estimates show that the contributions of charges of oxygen atoms and electric dipole moments of water molecules to EFG of Al and Na atoms are of the same order. Although mordenite structure differs from the structure of natrolite, this finding is consistent with our estimations of the various contributions to the EFG of Al and Na atoms in natrolite [31].

## Conclusion

Analysis of the temperature dependencies of ^27^Al and ^23^Na NMR spectra show that the shapes of the spectra are mainly determined by the second-order quadrupole interactions, while the dipole–dipole contribution coming from ^1^H nuclei is very small. The spin–lattice relaxation of ^27^Al and ^23^Na is governed by the electric quadrupole interaction of nuclei with the crystal electric field gradients modulated by translational motion of H_2_O molecules in the mordenite channels. The interaction of the ^27^Al and ^23^Na nuclear spins with paramagnetic impurities becomes to be significant relaxation mechanism at *T* < 200 K.
